# A polygenic score indexing a *DRD2*-related co-expression network is associated with striatal dopamine function

**DOI:** 10.1038/s41598-022-16442-6

**Published:** 2022-07-23

**Authors:** Enrico D’Ambrosio, Giulio Pergola, Antonio F. Pardiñas, Tarik Dahoun, Mattia Veronese, Leonardo Sportelli, Paolo Taurisano, Kira Griffiths, Sameer Jauhar, Maria Rogdaki, Michael A. P. Bloomfield, Sean Froudist-Walsh, Ilaria Bonoldi, James T. R. Walters, Giuseppe Blasi, Alessandro Bertolino, Oliver D. Howes

**Affiliations:** 1 Department of Psychosis Studies, Institute of Psychiatry, Psychology and Neuroscience, King’s College London, London, SE5 8AF UK; 2 Department of Basic Medical Sciences, Neuroscience and Sense Organs, University of Bari “Aldo Moro”, Bari, Italy; 3 Lieber Institute for Brain Development, Johns Hopkins Medical Campus, Baltimore, MD USA; 4 MRC Centre for Neuropsychiatric Genetics and Genomics, Division of Psychological Medicine and Clinical Neurosciences, School of Medicine, Cardiff University, Cardiff, UK; 5 Department of Child and Adolescent Psychiatry, Institute of Psychiatry, Psychology and Neuroscience, King’s College London, London, UK; 6 Department of Neuroimaging, Institute of Psychiatry, Psychology and Neuroscience, King’s College London, London, UK; 7 Department of Information Engineering, University of Padua, Padua, Italy; 8 Centre for Affective Disorders, Psychological Medicine, Institute of Psychiatry, Psychology and Neuroscience, King’s College London, London, UK; 9 Division of Psychiatry, University College London, 6th Floor, Maple House, 149 Tottenham Court Road, London, W1T 7NF UK; 10 Center for Neural Science, New York University, New York, USA; 11 Institute of Clinical Sciences (ICS), Faculty of Medicine, Imperial College London, Du Cane Road, London, UK; 12 H. Lundbeck A/S, Ottiliavej 9, 2500 Valby, Denmark

**Keywords:** Psychosis, Schizophrenia, Genetics of the nervous system

## Abstract

The D2 dopamine receptor (D2R) is the primary site of the therapeutic action of antipsychotics and is involved in essential brain functions relevant to schizophrenia, such as attention, memory, motivation, and emotion processing. Moreover, the gene coding for D2R (*DRD2*) has been associated with schizophrenia at a genome-wide level. Recent studies have shown that a polygenic co-expression index (PCI) predicting the brain-specific expression of a network of genes co-expressed with *DRD2* was associated with response to antipsychotics, brain function during working memory in patients with schizophrenia, and with the modulation of prefrontal cortex activity after pharmacological stimulation of D2 receptors. We aimed to investigate the relationship between the *DRD2* gene network and in vivo striatal dopaminergic function, which is a phenotype robustly associated with psychosis and schizophrenia. To this aim, a sample of 92 healthy subjects underwent ^18^F-DOPA PET and was genotyped for genetic variations indexing the co-expression of the *DRD2*-related genetic network in order to calculate the PCI for each subject. The PCI was significantly associated with whole striatal dopamine synthesis capacity (*p* = 0.038). Exploratory analyses on the striatal subdivisions revealed a numerically larger effect size of the PCI on dopamine function for the associative striatum, although this was not significantly different than effects in other sub-divisions. These results are in line with a possible relationship between the *DRD2*-related co-expression network and schizophrenia and extend it by identifying a potential mechanism involving the regulation of dopamine synthesis. Future studies are needed to clarify the molecular mechanisms implicated in this relationship.

## Introduction

The D2 dopamine receptor (D2R) is a G protein-coupled receptor coded by the *DRD2* gene and is involved in essential brain functions such as learning, memory, locomotion, attention, motivation, sleep, emotion processing, reproductive behaviour^1–3^. The D2R is also the primary site of the therapeutic action of antipsychotics^4–7^. Furthermore, one of the schizophrenia-associated *loci* from Genome-Wide Association Studies (GWAS)^8,9^ includes the D2R coding gene (*DRD2*), implicating this gene in the pathophysiology of schizophrenia^10^.

Genetic variations within *DRD2* have been associated with brain-related phenotypes, including working memory, sustained attention, variable attention control, emotion processing, dopamine binding in the striatum, suggesting that genetic mechanisms influence the effects of the D2R on brain function^11–17^. However, it is unlikely that genetic variations within a single gene explain the entire physiology related to specific brain phenotypes. In this regard, previous investigations have elucidated that genes involved in complex traits do not work in isolation but operate in networks of interacting genes^18–22^ acting via molecular pathways^23–25^. Genetic networks can be investigated in detail using methods for the analysis of gene co-expression patterns^26,27^. This approach is based on the evidence that the expression of different genes is influenced by common regulatory molecules, and that such gene expressions correlate^24,28–32^. Co-expressed genes are often related in terms of function^33,34^. A widely used technique to study gene co-expression is the weighted gene co-expression network analysis (WGCNA). WGCNA represents correlated gene expression into a graph that is designed to be scale invariant, hence reflecting the basic property of biological networks that include highly connected central hubs and more peripheral genes. Hierarchical clustering is used in WGCNA to define gene sets, called modules, that are tightly co-expressed. This approach has been used to identify, in post-mortem dorsolateral prefrontal cortex of healthy controls, a network of genes co-expressed with *DRD2*^35^, including genes associated with schizophrenia identified in the PGC2^9^ and PGC3 GWAS^36^. A follow-up study has supported with in vitro evidence the link between some of these genes and identified potential co-regulators^32^. Interestingly, a Polygenic Co-expression Index (PCI) predicting the brain-specific expression of this network of co-expressed genes was associated with response to antipsychotics and prefrontal inefficiency during working memory^35^, which has been consistently associated with schizophrenia^37^. Moreover, healthy subjects with higher PCI showed increased activation in the prefrontal cortex and longer reaction times when performing a working memory task^35^. Interestingly, in a recent network control theory study^38^ the same PCI has been shown to be related to dynamical brain state transitions during working memory in healthy volunteers. Furthermore, this PCI has been associated with within-subject variation of prefrontal cortex activity following pharmacological stimulation of D2R in a double-blind crossover design^39^.

While these studies focused on the frontal cortex, *DRD2* has its highest expression in the striatum^40^. Interestingly, molecular imaging studies show evidence that presynaptic striatal dopamine dysfunction plays an important role in abnormal reward processing and anomalies of other aspects of cognitive function^41,42^. Moreover, elevated striatal dopamine synthesis and release capacity are associated with schizophrenia^43–53^, psychotic symptoms^54^ and risk of psychosis^55,56^.

Whilst the findings discussed above show that the *DRD2* gene network is associated with cortical brain function relevant to cognitive phenotypes of schizophrenia^57^, it remains unknown if and how the genetic underpinnings of cortical dopaminergic function are related to striatal dopaminergic phenotypes associated with psychosis. The exploration of this relationship can be considered as particularly relevant in view of the connections between cortex and striatum^58^. The aim of the present study is to investigate the relationship between striatal dopamine synthesis capacity and co-expression of the *DRD2*-related genetic network^35^. To this aim, we analysed data from a cohort of healthy subjects that underwent ^18^F-DOPA PET and were genome-wide genotyped; we used the genetic variants indexing the co-expression of the *DRD2*-related genetic network to compute an individual PCI^35^. We hypothesised that higher PCI, which has been previously associated with greater prefrontal BOLD response (see also^59^) and longer reaction times during working memory processing^35^, would be associated with higher striatal dopamine synthesis capacity—thus outlining a consistent pattern of results resembling the physiological observations in patients with schizophrenia.

## Methods

### Participants

A total of 92 healthy subjects (demographics in Table 1) underwent ^18^F-DOPA PET scans^60^. The study was conducted in accordance with the Declaration of Helsinki and Good Clinical Practice. All participants gave informed written consent. The study was approved by the Administration of Radioactive Substances Advisory Committee (ARSAC), the South London and Maudsley/Institute of Psychiatry NHS Trust, the London Bentham Research Ethics Committee, and the Hammersmith Research Ethics Committee.

**Table 1:** Demographic characteristics of the sample.

	Total
N	92
Age (yr ± SD)	29.93 ± 8.84
Gender (male/female)	52/40
PET scanner (scanner 1/scanner 2/scanner 3)	37/35/20
K_i_^cer^ (1/min) whole striatum (mean ± SD)	0.0129 ± 0.0012
K_i_^cer^ (1/min) associative striatum (mean ± SD)	0.0128 ± 0.0012
K_i_^cer^ (1/min) limbic striatum (mean ± SD)	0.0130 ± 0.0014
K_i_^cer^ (1/min) sensorimotor striatum (mean ± SD)	0.0132 ± 0.0015
PCI (mean ± SD)	− 0.0085 ± 0.0986

Inclusion criteria were: age range 18–65 years, no history of major medical condition, good physical health. Exclusion criteria were: significant medical disorder or treatment, history of psychiatric illness (assessed using the Structured Clinical Interview for DSM-IV Axis I Disorders) including alcohol or substance abuse or dependence. The dataset has been gathered from our publicly available imaging data archive (https://maudsleybrc.nihr.ac.uk/research/precision-psychiatry/neuroimaging/neuroimaging-database-node/). The PET data have been previously published^61–65^, but the integration with the PCI has not been published before.

### Polygenic co-expression index

DNA was extracted from whole blood samples or cheek swabs using standard procedures^66^. Genome-wide genotyping was performed at Cardiff University, using HumanCore Exome 1.1 arrays ("Psych-chip", Illumina, San Diego, California, USA).

A PCI was calculated as previously described^35^. Briefly, a *DRD2* co-expression gene set, including 85 genes, was identified with a Weighted Genes Co-expression Network Analysis^67^ using the *post mortem* frontal cortex mRNA expression Braincloud database^68^. A set of 8 SNPs (*CHIT1* rs2486064, *GPLD1* rs6902039, *OSR1* rs851436, *POP1* rs9297283, *SDK2* rs1294071, *DHX33* rs1805453, *BTG4* rs1121391, *AGR2* rs1037791) associated with the first principal component of gene set co-expression was used to compute the PCI; a weight based on the co-expression profile of the gene set was assigned to each genotype of each SNP (Table S1). Genotyping was conducted for these SNPs. Genotype quality control for these SNPs was performed according to standard parameters^69^. Briefly, these included an individual missingness rate < 0.98, a SNP call rate > 0.98 and a Hardy–Weinberg equilibrium (HWE) *p* value > 10^−4^, as computed by the PLINK v1.9 software^70^.

### Population stratification

The Principal Components Analysis in Related samples (PC-AiR) method^71^ was used in R (GENESIS R/Bioconductor package^72^) on the full set of genotypes to generate the top 10 principal components of the sample, which were included as covariates of no interest in all the analyses, in order to correct for population stratification.

### PET scanning

^18^F-DOPA PET scans were performed to measure dopamine synthesis capacity (indexed as the influx rate constant K_i_^cer^)^73^.

### Image acquisition

Images were acquired in three-dimensional mode using three different PET scanners: an ECAT HR + 962 PET scanner (CTI/Siemens, Knoxville, Tennessee) and two Siemens Biograph HiRez XVI PET-CT scanners (Siemens Healthcare, Erlangen, Germany). After the administration of approximately 150 MBq of ^18^F-DOPA, dynamic PET data were acquired over a period of 95 min as previously described^61–64,74^.

### Image processing

The frames were aligned using a mutual information algorithm^75^. A movement-corrected dynamic image was then used in the analysis. A tracer-specific (^18^F-DOPA) template^76^ was normalised together with a striatal probabilistic atlas^77^ to the individual PET summation images. The influx constant (K_i_^cer^) for striatum was calculated using the cerebellum as a reference region^78^. For the exploratory analyses, the striatum was sub-divided into limbic, associative and sensorimotor parts on the basis of function and the topography of brain projections from limbic, associative and sensorimotor cortical areas to the striatum^48,77,78^.

### Statistical analysis

The effect of the PCI on whole striatal K_i_^cer^ was tested using a linear model (lm) regression in R^79^ with age, gender, PET scanner and the first 10 genetic principal components as covariates of no interest in view of their potential effect on dopamine synthesis capacity^80,81^. To facilitate the interpretation of the results, PCI values were standardised using the scale() function in R before being entered in the model^82^. Injected dose of radiotracer was not considered, as it is not associated with ^18^F-DOPA K_i_^cer^ estimates^74^. A significance threshold of α < 0.05 was used. Separate exploratory analyses were conducted to test the effect of the PCI on associative striatum, limbic striatum and sensorimotor striatum K_i_^cer^. R^79^ was used for all the statistical analyses. The R package ggplot2^83^ was used to plot the main results. To exclude the presence of outliers, the Rosner's test function ("rosnerTest") of the R package EnvStats^84^ was used to remove extreme observations.

## Results

Demographic (± SD) and K_i_^cer^ values included are reported in Table 1.

**Figure 1:**
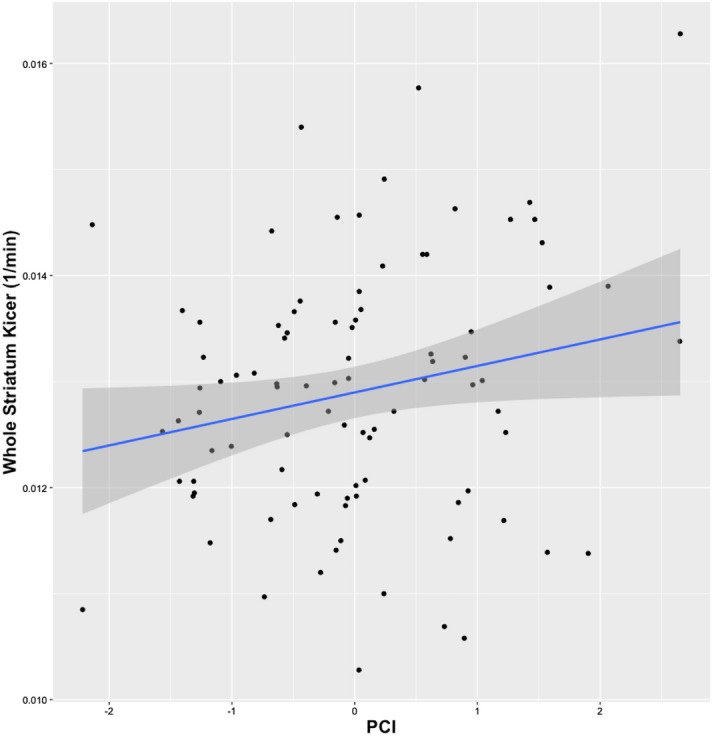
Scatterplot illustrating the correlation between whole striatum K_i_^cer^ (y axis) and PCI.

The Rosner's test did not reveal any outliers. PCI was significantly associated with whole striatal dopamine synthesis capacity (t value = 2.106, *p* = 0.038). Figure 1 illustrates a positive correlation between whole striatum K_i_^cer^ (y axis) and PCI. PET scanners, included as covariates of no interest, did not show a statistically significant association with dopamine synthesis capacity (t value = 1.603, *p* = 0.112).

**Figure 2:**
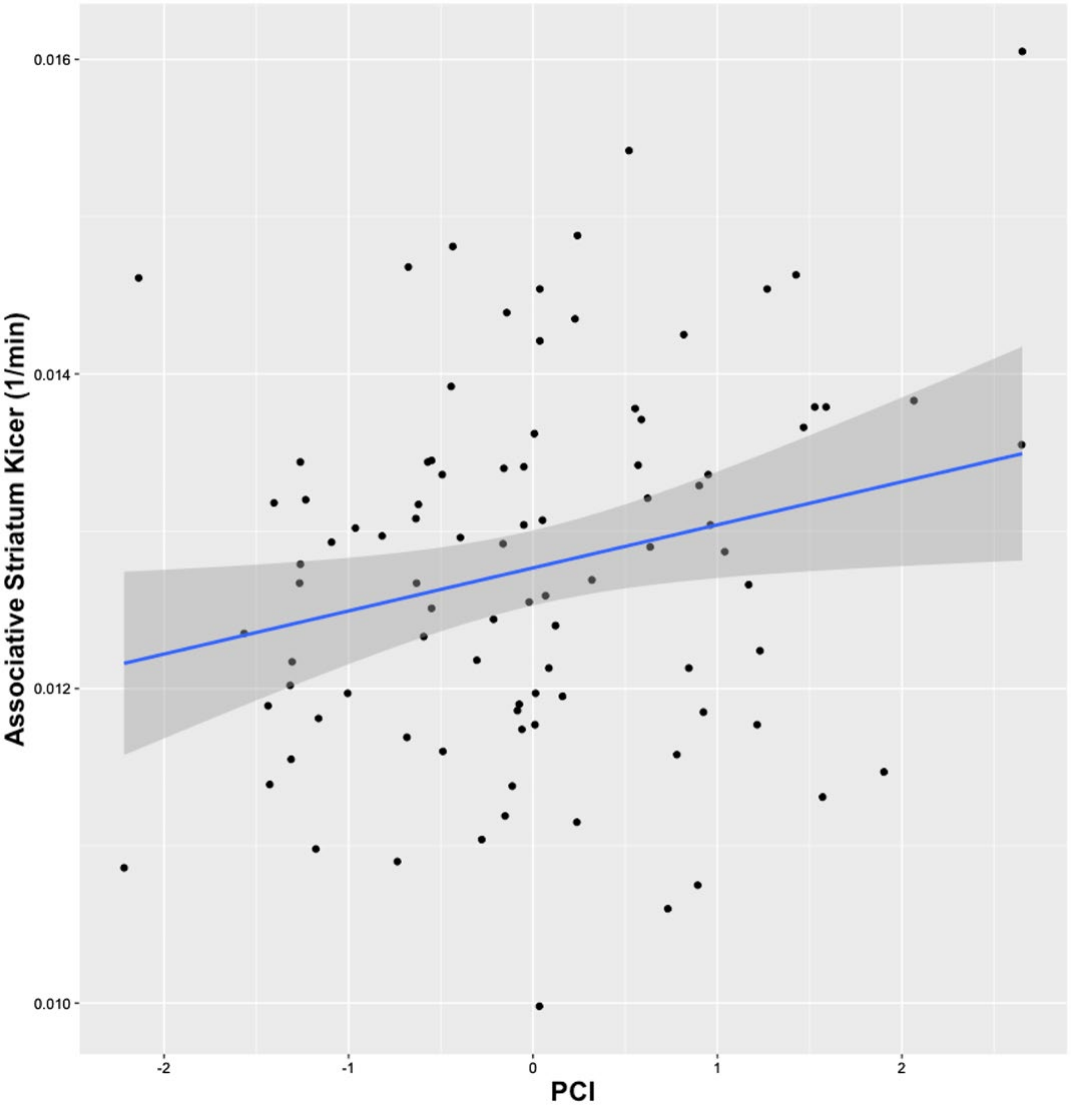
Scatterplot illustrating the correlation between associative striatum K_i_^cer^ (y axis) and PCI.

The exploratory analyses in the striatal subdivisions revealed an effect of the PCI on dopamine synthesis capacity for the associative striatum only (t value = 2.063, *p* = 0.042) (Fig. 2), while there was not a significant correlation with limbic striatum (t value = 1.957, *p* = 0.054) or sensorimotor striatum (t value = 1.841, *p* = 0.069). The interaction among striatal subdivision, PCI, and K_i_^cer^ was not significant (*p* = 0.738).

## Discussion

The present study shows for the first time an in vivo association between striatal dopamine synthesis capacity and a *DRD2*-related co-expression score in a cohort of healthy subjects. Specifically, high polygenic co-expression index, reflecting greater prefrontal co-expression of a *DRD2*-related genetic network, was associated with elevated striatal dopamine synthesis capacity. These results suggest that, besides *DRD2*, several genes and related products may be relevant to the modulation of striatal dopamine function.

Gene co-expression networks have been instrumental in identifying gene sets associated with antipsychotic treatment response^30,35^, phenotypes associated with schizophrenia^25,28,35,85^, clinical state and risk for schizophrenia^31^, and changes in prefrontal function after D2R stimulation^39^. As reviewed previously, increased dopamine synthesis capacity represents a phenotype associated with schizophrenia^47,86,87^. Thus, the results of the present study suggest a possible relationship between the *DRD2*-related co-expression network identified and schizophrenia. Consistently, the exploratory analyses in the different striatal subdivisions suggest that the PCI is associated with dopamine capacity in the associative striatum, which is the striatal region showing greater dopaminergic dysfunction in patients with schizophrenia compared with other striatal subdivisions according to meta-analytic evidence^88^. Nevertheless, it is important to note that the analyses in the different striatal subdivisions were only exploratory and there was no significant difference between effect sizes across striatal subdivisions. Additionally, the association with the associative striatum K_i_^cer^ would not survive correction for multiple comparisons. Therefore, these results should be considered as exploratory and require further evaluation in a larger sample.

The hypothesis of a positive relationship between PCI and striatal dopamine synthesis capacity was based on a study^35^ demonstrating in healthy subjects an association of this index with prefrontal inefficiency during working memory, another phenotype related to schizophrenia. However, it is noteworthy that previous studies have shown both positive^89–91^ and negative^92,93^ correlations between striatal dopamine synthesis capacity and working memory. Thus, a study investigating striatal dopamine function, working memory efficiency and PCI in the same sample would be necessary to elucidate the relationship between these factors.

Interestingly, fifteen genes of the *DRD2* Co-Expression Network (*ACR*, *ALDH3A1*, *BTN3A1*, *CALHM3*, *CES3*, *DRD2*, *EFCAB6*, *GALNT10*, *GATAD2A*, *GLI1*, *HIST1H1E*, *HIST1H3G*, *IL31*, *RBM6*, *SLC28A1*) are located within schizophrenia-associated loci in the latest Psychiatric Genetic Consortium investigation^36^. Notably, *GATAD2A* is among the genes resulting from the PGC3 prioritisation analysis due to its eQTL co-localisation profile^36^. Accordingly, it is considered a plausible causal gene for schizophrenia^94^. This gene codes for the protein GATA zinc finger domain containing 2A, a transcriptional repressor^95^, which is preferentially expressed during foetal brain development^96^. Its involvement in cell proliferation^97^ indicates a key role in development^94^. Furthermore, it has been implicated in schizophrenia through its involvement in the regulation of gene expression^98,99^. Consistently, it is upregulated in the hippocampus of patients with schizophrenia compared with healthy controls^94^.

Moreover, it has been recently demonstrated^32^ that the expression of genes of the *DRD2* co-expression module can be regulated by NURR1, a transcription factor regulating genes involved in the dopaminergic system^100^. As D2R is a potent NURR1 activator^101,102^, it has been hypothesised that antipsychotics, through the blockade of the D2R, can impact the expression of NURR1, which in turn can regulate the transcription of the genes included in the *DRD2* co-expression module^32^. The results of the present study—indicating a relationship between the *DRD2* co-expression network and an established phenotype linked to schizophrenia such as PET-estimated dopamine synthesis capacity—are consistent with the hypothesis of the involvement of the genes of this module in the pathophysiology of schizophrenia and mechanisms underlying the response to antipsychotics.

Notably, the approach used in the present study is data-driven and the genes within the network are not pre-defined; thus, the mechanisms through which the proteins coded by the genes of this network interact with the dopaminergic pathway still need to be clarified. In fact, it needs to be understood how the *DRD2* co-expression network influences striatal presynaptic dopamine synthesis capacity. In this context, it should be considered that post-synaptic D2 receptors play a role in the regulation of dopamine synthesis and release through inhibitory feedback loops^103,104^. It is also possible that the PCI reflects a different expression of the D2 autoreceptors, which regulate dopamine synthesis, although it should be noted that this score was developed analysing the expression of transcripts including exon 6, which is characteristic of the long isoform of D2R more often found post-synaptically^105,106^. Moreover, in view of the fact that the *DRD2* co-expression gene-set indexed by the PCI is enriched for “negative regulation of dopamine secretion (GO:0033602)”^35,39^, preclinical studies are needed to test the hypothesis that the transcriptomic context of *DRD2* influences dopamine presynaptic signalling.

Furthermore, in order to examine the potential involvement of this co-expression network in the regulation of expression and availability of the post-synaptic D2 receptors, it would be helpful to investigate the in vivo relationship between PCI and D2 receptor availability through studies using other PET tracers (e.g. ^11^C-raclopride).

The present study was conducted on healthy subjects; thus, the results were not influenced by medication or disease status. A key next step is thus to explore the effect of the *DRD2-*Polygenic Co-expression Index on dopamine function in disorders where involvement of the dopamine system has been demonstrated, such as psychosis, addiction, bipolar disorder^54,107,108^.

A potential limitation of the study is the use of data from three different PET scanners. However, the scanner was used as a covariate of no interest. Furthermore, we did not find a statistically significant association of PET scanner with K_i_^cer^; consistently, our recent investigation on the effect of the scanner in a similar dataset acquired from three different PET tomographs (Siemens Biograph 6 Hi-Rez, Siemens Biograph 6TruePoint, ECAT/EXACT3D) with an injected radioactivity below 200 MBq and acquisition time of 95 min did not reveal significant effects^109^.

Moreover, it should be considered that the *DRD2* co-expression pathway, and therefore the PCI used in this study, was calculated by using *post mortem* mRNA from the frontal cortex^35,68^, thus it would be interesting to test if the *DRD2* co-expression network remains the same in the striatum and the midbrain, where the dopamine neuron cell bodies are located.

Furthermore, in the present study, we did not examine dopamine function in the frontal cortex, due to lower ^18^F-DOPA signal reliability in frontal cortical regions when quantified without arterial blood input function^76,110^. Therefore, a study using PET tracers more suitable for the measurement of the cortical dopamine system^59,111^ would be helpful in understanding the relationships between PCI, striatal and cortical dopamine systems.

## Conclusions

The results from the present study indicate that a polygenic score indexing a *DRD2*-related co-expression network is associated with striatal dopamine function measured in vivo with ^18^F-DOPA imaging. Our findings suggest that the same genetic variants associated with prefrontal inefficiency during working memory are also associated with greater estimated dopamine synthesis in the striatum. In view of the hypothesised link between striatal hyperdopaminergia and prefrontal hypodopaminergia^59,112^, it is tempting to observe that these variants originally found analysing the prefrontal cortex may have more widespread system-level correlates^38^.

## Supplementary Information

Supplementary Table S1.

## Data Availability

The PET data are available in The NeurOimaging DatabasE (NODE) repository (https://maudsleybrc.nihr.ac.uk/research/precision-psychiatry/neuroimaging/neuroimaging-database-node/) upon request. The data^68^ used for the WGCNA performed to identify the *DRD2* co-expression gene set^35^ are available in the database of Genotypes and Phenotypes (dbGaP, https://www.ncbi.nlm.nih.gov/gap/, Study Accession: phs000417.v2.p1) and Gene Expression Omnibus (GEO, https://www.ncbi.nlm.nih.gov/geo/, Study Accession: GSE30272). The weights assigned to each genotype of each SNP are available in Supplementary Table S1.
