# Supplementary material for: A polygenic score indexing a DRD2-related co-expression network is associated with striatal dopamine function
Source: Sci Rep. 2022 Jul 23;12:12610. doi: 10.1038/s41598-022-16442-6 (PMC9308811; doi:10.1038/s41598-022-16442-6)
Supplement: Supplementary file 1 — Supplementary Table S1. [file 41598_2022_16442_MOESM1_ESM.docx]

**Table S1**

Weights assigned to each genotype of each SNP to calculate the PCI

| SNP | genotype | weight |
| --- | --- | --- |
| rs2486064 | CC | 0 |
| rs2486064 | CT | 0.00282005 |
| rs2486064 | TT | -1.0577694 |
| rs6902039 | CC | 1.38189949 |
| rs6902039 | CT | -0.136259 |
| rs6902039 | TT | 0 |
| rs851436 | AA | 0.06921098 |
| rs851436 | AC | 0.64032413 |
| rs851436 | CC | 0 |
| rs9297283 | GG | 0 |
| rs9297283 | GT | 0.47636775 |
| rs9297283 | TT | -0.750623 |
| rs12940715 | CC | 0 |
| rs12940715 | T-carriers | 0.56004884 |
| rs1805453 | AA | 0.7859023 |
| rs1805453 | AC | 0.12799484 |
| rs1805453 | CC | 0 |
| rs11213916 | CC | 0 |
| rs11213916 | CT | -0.4508729 |
| rs11213916 | TT | -0.6492373 |
| rs1037791 | AA | 0 |
| rs1037791 | AG | -0.1099644 |
| rs1037791 | GG | 0.70396625 |
